# Semisynthesis of site-specifically succinylated histone reveals that succinylation regulates nucleosome unwrapping rate and DNA accessibility

**DOI:** 10.1093/nar/gkaa663

**Published:** 2020-08-07

**Authors:** Yihang Jing, Dongbo Ding, Gaofei Tian, Ka Chun Jonathan Kwan, Zheng Liu, Toyotaka Ishibashi, Xiang David Li

**Affiliations:** Department of Chemistry, The University of Hong Kong, Pokfulam Road, Hong Kong, China; Division of Life Science, The Hong Kong University of Science and Technology, Clear Water Bay, Hong Kong, China; Department of Chemistry, The University of Hong Kong, Pokfulam Road, Hong Kong, China; Department of Chemistry, The University of Hong Kong, Pokfulam Road, Hong Kong, China; Department of Chemistry, The University of Hong Kong, Pokfulam Road, Hong Kong, China; Division of Life Science, The Hong Kong University of Science and Technology, Clear Water Bay, Hong Kong, China; Department of Chemistry, The University of Hong Kong, Pokfulam Road, Hong Kong, China

## Abstract

Posttranslational modifications (PTMs) of histones represent a crucial regulatory mechanism of nucleosome and chromatin dynamics in various of DNA-based cellular processes, such as replication, transcription and DNA damage repair. Lysine succinylation (Ksucc) is a newly identified histone PTM, but its regulation and function in chromatin remain poorly understood. Here, we utilized an expressed protein ligation (EPL) strategy to synthesize histone H4 with site-specific succinylation at K77 residue (H4K77succ), an evolutionarily conserved succinylation site at the nucleosomal DNA-histone interface. We then assembled mononucleosomes with the semisynthetic H4K77succ *in vitro*. We demonstrated that this succinylation impacts nucleosome dynamics and promotes DNA unwrapping from the histone surface, which allows proteins such as transcription factors to rapidly access buried regions of the nucleosomal DNA. In budding yeast, a lysine-to-glutamic acid mutation, which mimics Ksucc, at the H4K77 site reduced nucleosome stability and led to defects in DNA damage repair and telomere silencing *in vivo*. Our findings revealed this uncharacterized histone modification has important roles in nucleosome and chromatin dynamics.

## INTRODUCTION

Nucleosome is the fundamental unit of DNA packaging in eukaryotic cells, composed of two copies of histone H2A, H2B, H3 and H4 in an octameric complex, wrapped by 147 base pairs of duplex DNA (1,2). The nucleosomal structure dynamically disassembles and assembles to regulate accessibility to the DNA during various cellular processes, such as gene expression, DNA replication and DNA damage repair (3–5). The posttranslational modifications (PTMs) of histones stand out as one of main mechanisms for regulating nucleosome stability and dynamics during every distinct step in the organization of chromatin, including histone folding, assembly of DNA and histones into nucleosomes, and compaction of nucleosomes into higher-order structures of chromatin (5).

Various types of PTMs have been identified on histones, such as acetylation, methylation, phosphorylation and ubiquitinylation (6). These PTMs can often affect both inter- and intra-nucleosomal interactions, as they alter the physical and chemical properties of the modified residues. One of the most studied histone PTMs is lysine acetylation (Kac), which neutralizes the positively charged lysine side chain under physiological conditions. It has been shown that acetylation at histone H4K91 residue, which is located at the histone H2A–H2B dimer and (H3-H4)_2_ tetramer interface, impairs nucleosomal histone interactions and thus destabilizes the nucleosome (7). Acetylation at H3K56 residue, a site at the DNA entry/exit region of the nucleosome, influences histone–DNA interaction and increases DNA breathing and accessibility (8–10).

In addition to acetylation, a variety of other lysine acyl modifications on histones have been recently discovered, including lysine malonylation (Kmal), succinylation (Ksucc), and 3-hydroxyl-3-methylglutarylation (HMG-K) (11–14). Ksucc was initially discovered in mitochondrial proteins involved in the TCA cycle, fatty acid metabolism, and amino acid degradation, which imply Ksucc potentially has roles in diverse metabolic pathways (15). It has been found that SIRT5, a nicotinamide adenine dinucleotide (NAD)-dependent deacetylase in sirtuin family, primarily functions as the desuccinylase that catalyzes the removal of succinylation from lysine residues (15). Although succinyl-CoA can non-enzymatically modify substrates *in vitro*, very recent studies have revealed that succinylation can be catalyzed by succinyltransferase using succinyl-CoA as a substrate in cells. For example, lysine acetyltransferase 2A (KAT2A, also known as GCN5) can specifically succinylate histone H3K79 to promote tumor development (16). More recently, carnitine palmitoyltransferase (CPT) 1A was demonstrated to function as a novel succinyltransferase *in vivo* and *in vitro* (17). Emerging clinical evidence indicates that Ksucc dysregulation is associated with the progression of various diseases, including cancers and neurodegeneration disorders (18,19).

Compared with Kac, Ksucc has a negatively charged carboxylate group that results in more distinct chemical and structural changes in lysine residues. However, it remains unknown whether the change in charge status (from +1 to −1) directly influences nucleosomal structure and dynamics due to disrupted DNA–histone interactions. Indeed, Lys-to-Glu mutations, which mimic Ksucc, at a couple of histone Lys residues leads to defects in the chromatin structure in budding yeast (13). However, despite distinct differences between Glu and Ksucc, direct experimental evidence supporting this hypothesis is still lacking and the roles played by negatively charged histone acylations in regulating chromatin-associated processes remain poorly understood.

To fill this knowledge gap, we have developed a method for site-specific installation of a Ksucc mimic onto histones (20). The incorporation of the Ksucc mimic at histone H2BK34 decreased nucleosome stability and influenced dynamic processes of nucleosome assembly and disassembly. Notably, besides H2BK34, multiple succinylated lysine residues also lie at the DNA–histone interface of nucleosomes, which may affect nucleosome and chromatin dynamics. The K-to-E mutation (mimic of Ksucc) at histone H4K77 site in budding yeast causes several phenotypes consistent with chromatin structure defects (13). Although H2BK34 and H4K77 residues are both located at the DNA–histone interface, H4K77 residue reside in a more inner position of the nucleosome close to the nucleosome dyad (Supplementary Figure S1). On the other hand, simultaneous acetylation at histone H4K77 and K79 residues promoted nucleosome unwrapping (9). However, acetylation of H4K77 alone was suggested to result in repressed chromatin structure (21).

In the present study, we focused on H4K77succ to examine whether and how this Ksucc may affect nucleosome structure and dynamics. Importantly, instead of using a Ksucc mimic, we prepared the actual succinylated histone using the expressed protein ligation (EPL) strategy to profile the specific regulatory roles of H4K77succ in nucleosome and chromatin dynamics (22). A variety of biochemical and biophysical characterizations of the H4K77succ-containing nucleosomes suggest that this negatively charged modification indeed influences nucleosome dynamics by facilitating DNA unwrapping at the nucleosome entry-exit region to increase DNA accessibility.

## MATERIALS AND METHODS

### General methods and materials

All solvents used are dried and distilled by using standard methods, and all the chemical reagents and amino acids for peptide synthesis without further noted were purchased from Sigma-Aldrich and GL Biochem. Unless otherwise noted, all the purchased chemicals were used without further purification. Unless otherwise noted, all the media and reagents used for yeast experiments were purchased from Formedium. Antibodies were purchased from Abcam (United Kingdom) (anti-histone H2B (yeast), 1:2000, ab188291) and Santa Cruz Biotechnology (USA) (goat anti-rabbit IgG-HRP, sc-2004, 1:10 000). For the purification of peptides and proteins by high performance liquid chromatography (HPLC), the mobile phases are buffer A (100% water, 0.1% TFA) and buffer B (90% ACN in water, 0.1% TFA).

### Peptide synthesis

The unnatural amino acid Fmoc-Lys(*t*Bu–Succinyl)-OH used for peptide synthesis was synthesized as reported previously (20). The peptide used in this research was synthesized on 2-chlorotrityl chloride resin following standard Fmoc-based solid-phase peptide synthesis protocol. After the coupling of all amino acids, the removal of protecting groups and cleavage of peptides from the resin were done by incubating the resin with cleavage cocktail containing 95% trifluoroacetic acid (TFA), 2.5% triisopropylsilane, 1.5% water and 1% thioanisole for 2 h. Peptides were purified on a preparative high performance liquid chromatography (HPLC) system (Waters 2535 Quaternary Gradient Module, Waters 515 HPLC pump) with a Vydac C18 column (22 mm × 250 mm, 10 μm, Garce). The purity (>95%) and identity of peptide was confirmed by LC–MS.

### Preparation of H4K77succ peptide

**Figure 1.**
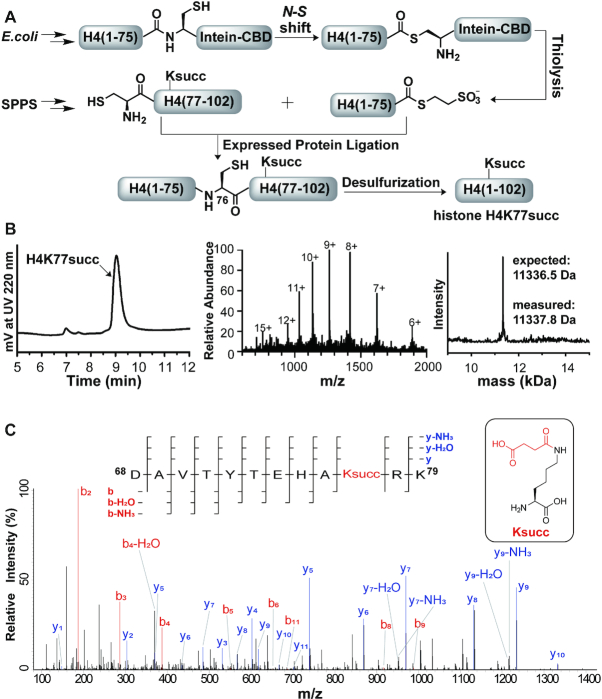
Semisynthesis of histone H4K77succ by expressed protein ligation (EPL). (**A**) EPL strategy for constructing histone H4K77succ. (**B**) LC–MS and deconvolution results of the synthesized full-length histone H4K77succ. (**C**) Higher-energy collisional dissociation tandem MS spectra of a Ksucc-incorporated histone H4 at K77, ^68^DAVTYTEHAKsuccRK^79^.

The chemical synthesis of succinylated histones H4K77succ was achieved by using the EPL strategy (22). Synthesis of histone H4K77succ by EPL requires synthetic Ksucc peptide containing an N-terminal cysteine and recombinant proteins carrying a C-terminal thioester. We synthesized the H4 peptide (76-102, A76C) with succinylation at K77 (Supplementary Figure S2). At the same time, we expressed and purified the recombinant H4 (1–75) in fusion with an intein and a chitin-binding domain (CBD) from inclusion bodies. The purified H4 (1–75)-intein-CBD was then refolded by dialyzing into a high salt buffer (1 M NaCl, 25 mM HEPES, pH 7.5, 1 mM EDTA) at 4°C. For the ligation reaction, H4 (1–75)-intein-CBD protein was first reacted with sodium 2-mercaptoethanesulfonate (MESNa) to afford the required C-terminal thioester, which was then incubated with the synthetic peptide. After EPL, radical-induced desulfurization was applied to convert the cysteine back to alanine (Supplementary Figure S3). The final product H4K77succ was purified by preparative HPLC. The purity and identity of ligated H4K77succ was checked by LC-MS (Figure 1A and B).

### Preparation of *Xenopus laevis* histones and LexA protein

All the recombinant histones (H1.4, H2A, H2B, H3, H4, H2A K119C, H2B T112C) were expressed and purified as previously described (23). Mutations of H2A K119C and H2B T112C were introduced by site-directed mutagenesis. The primers for mutagenesis were listed in Supplementary Data. LexA protein was expressed and purified from the pJWL288 plasmid, gift from Prof. Michael G. Poirier (Department of Physics, Department of Chemistry & Biochemistry, Ohio State University), as previously described (24).

### Generation of fluorophore labeled histone H2A and H2B

To generate Alexa Fluor 488 labeled histone H2B (H2B-Alexa488), histone H2B with T112C mutation was dissolved in reaction buffer (25 mM HEPES, pH 7.5, 6 M guanidine, 5 mM TCEP) to a final concentration of 0.5 mM. Then five molar equivalents of Alexa-488-C_5_-maleimide (Invitrogen) was added to the reaction mixture. Keep the reaction for 4 h at 37°C. The fluorophore labeled histone was purified by HPLC using preparative C4 column (25 mm × 250 mm, 10 μm, Grace). The purity and identity of the product was confirmed by LC–MS. Deconvolution result was obtained by UniDec software (25) (Supplementary Figure S4). Same method was applied to generate Cy5 (Cy5 Maleimide Mono-reactive Dye, GE) labeled histone H2A at K119 position (Supplementary Figure S5).

### Sample preparation for mass spectrometry

The semisynthesized H4K77succ protein was dissolved in lithium dodecyl sulfate sample loading buffer (Life Technologies) with 50 mM dithiothreitol (DTT), heated at 75°C for 8 min, and then reacted with iodoacetamide in dark for 30 min to alkylate all the reduced cysteines. Proteins were then separated on a Bis–Tris gel, followed by fixation in a 50% methanol/7% acetic acid solution. The gel was stained by GelCode Blue stain (Pierce). The gel slice containing H4K77succ protein was cut off and diced to 1 mm cubes, then destained with 50 mM ammonium bicarbonate/50% acetonitrile for 1 h. The destained gel cubes were dehydrated in acetonitrile for 10 min and rehydrated in 25 mM ammonium bicarbonate with trypsin for protein digestion at 37°C overnight. The resulting peptides were enriched with StageTips. The peptides eluted from the StageTips were dried down by SpeedVac and then resuspended in 0.5% acetic acid for analysis by LC–MS/MS (Figure 1C).

### Preparation of histone H2A–H2B dimer and (H3–H4)_2_ tetramer

The preparation of histone dimer and tetramer used the same method as previously reported(23). Briefly, to obtain H2A–H2B dimers, equal amount of H2A and H2B (or fluorophore labeled H2A and H2B) was mixed and dissolved in unfolding buffer (6 M guanidine, 10 mM Tris–HCl, pH 7.5, 1.0 mM EDTA, 10 mM DTT) to a final histone concentration of 1 mg/mL. The mixture was incubated on ice for 30 min and followed by dialysis into histone refolding buffer (2 M NaCl, 10 mM Tris–HCl, pH 7.5, 1.0 mM EDTA, 5 mM 2-mercaptoethanol) at 4°C. The refolded dimers were then purified over a Superdex 200 pg column (HiLoad 16/60, GE). Same method was applied to prepare with or without fluorophore labeled (H3–H4)_2_ tetramer.

### Preparation of fluorophore labeled DNA fragments

The fluorophore labeled DNA fragments were prepared by PCR using primers labeled with Alexa 488, Alexa 594 or Cy3 as previously reported (9,20,26,27). The template for Cy3-labeled DNA was the ‘Widom 601’ DNA with the 8th–27th base pairs replaced with the LexA recognition sequence (TACTGTATGAGCATACAGTA).

The PCR products were precipitated by isopropyl alcohol, washed by ethanol and dissolved in water and then purified by size-exclusion column (GE Superose 6 increase 10/300). The DNA concentration was determined by UV–Vis spectrophotometer (260 nm, NanoDrop 2000, Thermo).

### Preparation of nucleosomes for EMSA assay

Histone dimer and tetramer were assembled into nucleosomes with DNA by step-wise dialysis as described in previously reported (28). Briefly, purified histone H2A-H2B dimer and tetramers (i.e. H3–H4K77succ)_2_ or (H3–H4)_2_) were incubated with 153 bp of SELEX-generated ‘Widom 601’ DNA (the molar ratio is dimer:tetramer:DNA = 2:1:1) at concentration of 2 μM in 50 μl reconstitution buffer (2 M KCl, 10 mM Tris–HCl, pH 7.8, 0.1 mM EDTA). The samples were transferred into a Slide-A-Lyzer MINI dialysis unit (Thermo) and dialyzed at 4°C against reconstitution buffer containing 1.6 M KCl, 1.4 M KCl, 1.2 M KCl, 1.0 M KCl, 0.8 M KCl, 0.5 M KCl, 0.2 M KCl for 50 min each, and followed by 10 mM KCl for overnight. Same method was used to prepare DNA–(H3–H4)_2_ tetrasomes. The reconstituted tetrasome and nucleosomes were then resolved on native-PAGE (5% TBE gel, acrylamide/bis-acrylamide, 29:1). The gel was stained with ethidium bromide (EB) for 15 min and nucleosomes were visualized by UV with a MyECL Imager system (Thermo Fisher Scientific).

### Preparation and purification of fluorophore-labeled nucleosomes

Dimers and tetramers were incubated with ‘Widom 601’ DNA (the molar ratio is dimer:tetramer:DNA = 4:1:1, the excess amount of dimers help to generate fully compacted nucleosome) at a concentration of 2 μM in 50 μl reconstitution buffer. The dialysis procedure is the same as that for nucleosomes described above. The reconstituted nucleosomes were then purified by a native-PAGE (5% TBE gel, acrylamide/bis-acrylamide, 60:1) using mini prep cell (Bio-rad, Model 491). The purity of nucleosomes used for FRET assay was checked by a native-PAGE (Supplementary Figure S7a).

### FRET efficiency measurements for salt-induced nucleosome destabilization process

To measure salt-induced change in nucleosome stability, the nucleosomes were labeled with fluorophores at various positions (Supplementary Figure S7b). The donor and acceptor for FRET are Alexa Fluor 488 (ex/em 490/525 nm) and Alexa Fluor 594 (ex/em 590/617 nm), respectively. Freshly prepared pure fluorophore labeled nucleosomes were added into FRET buffer (10 mM Tris, 0.1 mM EDTA, pH 7.5, 1 mM ascorbic acid and 0.1 g/L BSA) containing different salt concentrations from 0.01 to 1.9 M. Then, the nucleosomes were incubated in the 384-well microplates. The fluorescence intensities were taken by plate reader (Victor X5, PerkinElmer) for three times. Energy transfer changes caused by salt-induced nucleosome dissociation were measured by emission of the acceptor upon selective donor excitation as previously described (20,26,27).

### FRET measurements of nucleosomal DNA unwrapping by LexA titration

The accessibility of DNA at entry-exist region was determined from the reduction in FRET efficiency as LexA binds to its target site in the nucleosome (8,9,29). Briefly, 10 nM Cy3–Cy5 labeled nucleosomes were titrated with LexA protein from 0 to 100 μM in 0.5× TE buffer containing 75 mM NaCl and incubated for 10 min. Cy3–Cy5 labeled nucleosomes were excited by using 531/25 nm bandpass filter for Cy3 and 635/25 nm bandpass filter for Cy5. The acceptor (Cy5) emission was collected by using 670/25 nm bandpass filter. For each sample, fluorescence intensity was taken three times by plate reader (Victor X5, PerkinElmer). The measurement of FRET efficiency was calculated by the (ratio)_A_ method that previously reported (30). The (ratio)_A_ equation: ${\rm{E}} = 2[( {\frac{{{\epsilon ^A}(v^{\prime\prime}){F^A}(v^{\prime})}}{{{F^A}(v^{\prime\prime})}} - {\varepsilon ^A}(v^{\prime})} ]/[{\varepsilon ^D}(v^{\prime}){d^ + }]$. The normalized FRET efficiencies upon LexA concentrations were fit to a non-competitive binding curve, *E* = *E*_0_ + (*E*_F_ − *E*_0_) / (1 + *S*_1/2_ / [LexA]), where *E* is the normalized FRET efficiency at a concentration of LexA protein; *E*_0_ is the normalized FRET efficiency in the absence of LexA protein and *E*_F_ is the normalized FRET efficiency at high concentration of LexA protein; *S*_1/2_ is the LexA concentration at which the normalized FRET efficiency has been reduced by half.

### ‘One-pot’ assay

The ‘one-pot’ assay was performed following the reported method (31,32). Briefly, the 601.2 nucleosomal DNA sequences bearing HaeIII and EcoRI digestion sites at indicated sites (Figure 3F and Supplementary Figure S10a) was purchased from BGI. The eight 601.2 DNA sequences were mixed in equal amounts and followed by ^32^P-labeling at ends of each 601.2 DNA. Then, the ^32^P at 3′-end was removed by EcoRI digestion. The ^32^P-labeled DNA was mixed with cold DNA in a 1:10 molar ratio. Then the nucleosome was loaded by stepwise dialysis. The HaeIII enzyme digestion (final concentration at 1.5 U/μL, NEB R0108S) was carried out in digestion buffer (10 mM Tris, pH 7.9, 10 mM MgCl_2_, 150 mM NaCl, 20 μg/mL BSA) with 100 nM nucleosome at 37 °C. Same amount of sample was removed at 0, 5, 10, 20, 40, 60 min time point, and the aliquot part reaction was stopped by adding proteinase K in reaction stop buffer (120 mM EDTA, 0.4% SDS) and kept at 37°C for 20 min. The final product was analyzed with 8% native-PAGE (acrylamide/bis-acrylamide, 60:1) in 1× TBE buffer. The same amount of ^32^P-labeled DNA mixture total digestion product was loaded for quantification. To compare the HaeIII digestion efficiency on unmodified and H4K77succ nucleosomes at each HaeIII position, the intensities of the unmodified nucleosome digested DNA fragments were normalized to 100%, and the intensity of the H4K77succ nucleosome digested DNA fragments were compared accordingly.

### Single molecule optical tweezers analysis

The nucleosomes for optical tweezers assay were reconstituted from histone octamer and 258 bp ‘Widom 601’ DNA with adding linker histone H1.4 as previously reported (28). The reconstituted nucleosomes were further purified by native-PAGE (5% TBE gel) using mini prep cell (Bio-rad). Two DNA ends of a single nucleosome were ligated to a 0.8 kb DNA fragments labeled with biotin or a 0.5 kb DNA fragment labeled with digoxygenin (BGI), respectively. The biotin and digoxygenin on nucleosomal DNA allowed formation of a DNA tether containing a single nucleosome between the streptavidin-coated bead (SA) and antidigoxigenin-coated bead (AD) held in optical traps.

To monitor the force extension curve on nucleosome, the force was increased by stepping the trap in 200 nm/s with the buffer (20 mM Tris, pH 7.5 and 40 mM NaCl). The data were collected at 200 Hz and decimated to 50 Hz to extract the outer and inner unfolding forces. To extract the nucleosome wrapping and unwrapping rates at outer rip, the beads were held at constant positions with the buffer (10 mM Tris, pH7.5 and 5 mM NaCl). The data were collected at 1 kHz and decimated to ∼250 Hz. Transitions were determined by running a *t*-test analysis between two adjacent windows of the wrapping/unwrapping traces.

The energy barrier of the outer wrap was extracted by the methods shown in previous literature (33–35). Briefly, the free energy Δ*G°* of the nucleosome outer DNA wrap was calculated based on the following formula:

$$\begin{equation*}\Delta {G^{\rm o}} = {F_{{\rm eq}}}\Delta x - \Delta {G_{{\rm strech}}} - {k_{\rm B}}T\,{\rm{ln}}\,{K_{{\rm eq}}}\end{equation*}$$

The equilibrium force (*F*_eq_) was obtained from the force at which the wrapping rate (*k*_w_) equals to the unwrapping rate (*k*_u_). The Δ*x* is the extension change during the unwrapping and the rewrapping. Δ*G*_stretch_ is the energy needed to stretch the DNA template to the *F*_eq_. The equilibrium rate (*K*_eq_) is *k*_w_ or *k*_u_ at *F_eq_*.

### Yeast strains construction

Saccharomyces cerevisiae YPH499 (*MAT a met15-Δ0 ura3-52 lys2-801 ade2-101 his3-Δ200 trp1-Δ63 leu2-Δ1*) was the principal strain background used throughout the work. Yeast strains were routinely cultured at 30°C with rotary aeration in YPD broth (1% yeast extract, 2% peptone, 2% glucose) or in YPD agar plate (YPD plus 2% (w/v) agar).

A list of yeast strains used for this work is supplied in Supplementary Table S2. The point mutations of histone H4 lysine (K) 77 site were introduced into HHF1 gene (one of the two gene copies that coding H4 protein in yeast) by a two-step method reported before (36) to afford XDL19 (HHF1 K77E), XDL20 (HHF1 K77Q) and XDL21 (HHF1 K77R). Then the nonessential HHF2 gene (the other gene copy that coding H4 protein in yeast) deletions were done by a PCR based gene depletion method to generate yeast strain XDL23 (H4 K77E), XDL24 (H4 K77Q), XDL25 (H4 K77R) (37).

XDL18 and XDL27-29 strains with URA3 inserted adjacent VR telomere were constructed by transforming YPH499 and XDL23-25 yeast with EcoRI cleaved pV-R URA3-TEL plasmid fragment. XDL16 and XDL31-33 strains with URA3 inserted adjacent VII-L telomere were constructed by transforming YPH499 and XDL23–25 yeast with EcoRI and SalI cleaved pVII-L URA3-TEL plasmid fragment. All the transformants were selected as being Ura+.

### Yeast spot assay

Tenfold serial dilutions of the cell suspensions were prepared, and 3 μl of each diluted suspension was spotted onto YPD plate (1% yeast extract, 2% peptone, 2% glucose, 2% (w/v) agar) or YPD plate plus 100 mM hydroxyurea (HU) plate. The plates were incubated at 30°C for 3 days and then photographed. For thermotolerance assay, the plates were incubated at 37°C for 3 days.

### Telomeric silencing assay

Tenfold serial dilutions of the cell suspensions were prepared, and 3 μl of each diluted suspension was spotted onto synthetic complete plate (SC), synthetic complete plate without uracil (SC-ura) and synthetic complete plate containing 0.1% 5-fluoroorotic acid (SC + 5-FOA). The plates were incubated at 30°C for 3 days and then photographed.

### Hydroxyapatite chromatography

Yeast chromatin was extracted as reported before (38). Extracted chromatin was resuspended in buffer A (10 mM NaCl, 10 mM Tris–HCl, pH 8.0, 10 mM sodium butyrate, 1 mM EDTA, 0.5 mM phenylmethylsulfonyl fluoride (PMSF) and EDTA-free protease inhibitor cocktail (cOmplete™, Roche)) and sonicated on ice. The clear supernatant from ultracentrifugation was incubated with hydroxyapatite beads (Sigma) at 4°C for 30 min. After washing with buffer B (50 mM sodium phosphate, pH 6.8, 10 mM sodium butyrate, 0.5 mM PMSF and EDTA-free protease inhibitor cocktail) containing 0.15 M NaCl, the hydroxyapatite beads were equally aliquoted. Then the beads were eluted with buffer B at NaCl concentrations from 0.25 to 0.8 M, respectively. The eluted and bounded fractions at each NaCl concentration were collected and resolved by SDS-PAGE. H2B protein was probed by anti-yeast H2B antibody (Abcam, ab188291). The intensity of H2B signal in Western blot were quantified by ImageJ.

A sigmoidal function below was fitted to the experimental curves. The stabilities of extracted chromatin were quantified by the ${C_{1/2}}$ parameter, namely the salt concentration where the intensity (Y axis) has dropped to half its initial value.

$$\begin{equation*}{{Y}}\left( {{X}} \right) = {{Y}}\left( {{\rm min}} \right) + \ \frac{{{{Y}}\left( {{\rm max}} \right) - {{Y}}\left( {{\rm{min}}} \right)}}{{1 + \exp \left( {{C_{1/2}} - \ {{X}}} \right)/b\ }}\end{equation*}$$

Here, *X* is the salt concentration in molar (M), and *b* describes the slope of the curve at *X* = ${C_{1/2}}$. *Y*(max) and *Y*(min) are min and max values in *Y* axis, respectively.

## RESULTS AND DISCUSSION

### Semisynthesis of histone H4K77succ

To study histone PTMs *in vitro* requires the generation of homogeneous histones that carry the site-specific modifications at a stoichiometric level. We therefore utilized the EPL strategy to synthesize site-specifically succinylated histone H4 at K77 for subsequent biochemical and biophysical studies (Figure 1A). Specifically, the truncated histone fragment H4(1–75) was fused to an intein-CBD tag and this recombinant protein was then expressed in *Escherichia coli*. After protein purification and refolding, thiolysis of the intein-CBD tag resulted in the H4(1–75) α-thioester, which was subsequently ligated to a synthetic peptide succinylated at H4K77 using Ala76Cys for the ligation. After desulfurization and protein purification, we obtained homogeneously succinylated histone H4 (H4K77succ). The purity and identity of this semisynthetic histone was confirmed by analytical liquid chromatography–mass spectrometry (LC–MS) (Figure 1B). The LC–tandem MS analysis of the tryptic digested H4K77succ confirmed the site-specific installation of Ksucc on the protein (Figure 1C).

### H4K77succ destabilizes the nucleosome and promotes H2A-H2B dimer release

We next used the semisynthesized histone H4K77succ to reconstitute mononucleosomes *in vitro*. We first introduced H4K77succ into (H3-H4)_2_ tetramers, and then combined this with unmodified H2A–H2B dimers and DNA encoding ‘Widom 601’ positioning sequence to assemble mononucleosomes (Supplementary Figure S6a) (39). The reconstituted mononucleosomes were examined by a gel electrophoresis mobility shift assay (EMSA), in which differences in the masses of the particles would be revealed by their inversely proportional mobility. As shown in Supplementary Figure S6b, the incorporation of succinylated H4 at K77 led to the formation of a larger proportion of (H3–H4)_2_ tetramer–DNA complex (i.e. tetrasome), whereas the unmodified core histones predominantly assembled into intact mononucleosomes. This result suggests that H4K77succ can perturb nucleosome assembly. Considering that K77 faces toward the DNA in the nucleosome, we hypothesize that H4K77succ might destabilize the nucleosome by weakening DNA–histone interactions.

**Figure 2.**
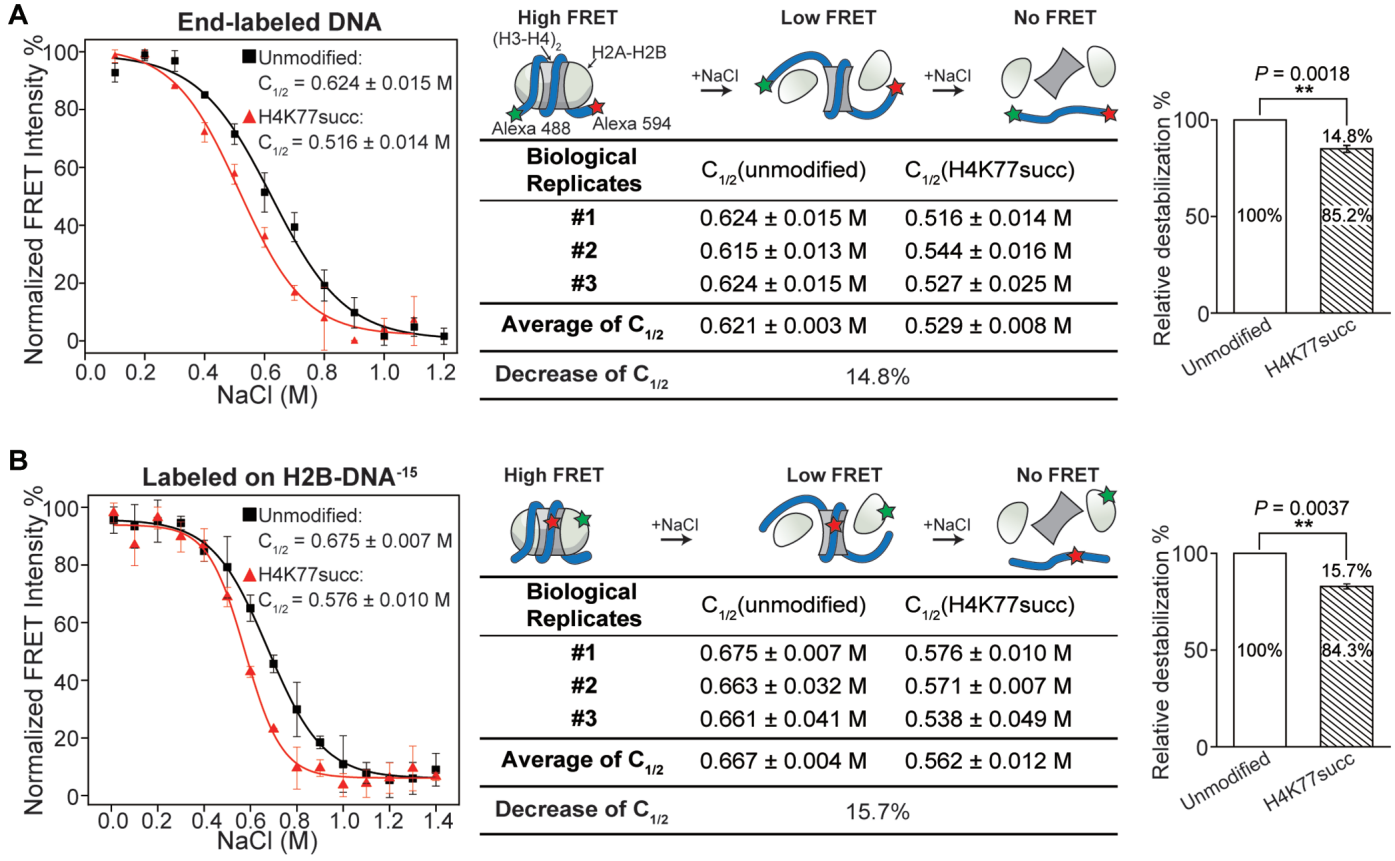
FRET-based approach for studying the effects of histone H4K77succ on nucleosome stability. Plot shows the normalized FRET intensity versus salt concentration in unmodified (black trace) and H4K77succ (red trace) nucleosomes labeled by FRET pairs at DNA ends (**A**) and H2B-DNA^−15^ (**B**). The salt concentration at which the FRET decreases by 50% is denoted as *C*_1/2_. For visualization, all curves were normalized between 100% at the maximum FRET value and 0 at high salt concentration. Left panel, a representative salt titration plot of the unmodified and H4K77succ nucleosomes. Salt titrations were repeated three times on nucleosomes from one preparation (*n* = 3, *C*_1/2_ = mean ± s.e.m). Middle panel, the cartoon indicates the relative locations of labels on the nucleosome and the process of salt-induced nucleosome dissociation. The *C*_1/2_ values of independent experiments in each type of nucleosome are showed. The average of *C*_1/2_ (mean ± s.e.m.) was calculated from three biological replicates (independent nucleosome preparations). The decrease in *C*_1/2_ value was calculated as $( {{{{C}}_{1/2}}( {{\rm{unmodified}}} ) - {{{C}}_{1/2}}( {{\rm{H}}4{\rm{K}}77{\rm{succ}}} )} )/{{{C}}_{1/2}}( {{\rm{unmodified}}} ) \times {\rm{\ }}100{\rm{\% }}$. Right panel, histogram shows the change in nucleosome stability associated with succinylation modification. The mean of the C_1/2_ values of unmodified nucleosomes was set to 100%. The *P* values were based on a two-tailed Student's *t* test. **P < 0.01.

To test this hypothesis, we used a Förster resonance energy transfer (FRET)-based assay to examine nucleosome stability. It is well known that a nucleosome disassembles in response to increasing ionic strength in a stepwise manner, involving the dissociation of the H2A–H2B dimers from the nucleosome, followed by the final release of the (H3–H4)_2_ tetramer from DNA (27). The structural transitions during this nucleosome disassembly process can be monitored by labeling the nucleosome with a pair of FRET dyes at various locations, and Supplementary Figure S7b shows the crystal structure of a nucleosome with FRET labeling at different positions (26,27,40–42). To evaluate the effect of H4K77succ on nucleosome stability, we first assembled and purified unmodified and H4K77succ nucleosomes, in which the ends of the nucleosomal DNA were labeled with Alexa 488 and Alexa 594 as donor and acceptor fluorophores, respectively (Figure 2A). The close proximity of the DNA ends in a fully assembled nucleosome enables efficient FRET, which is lost when the DNA ends are loosened from the nucleosome during the dissociation of the H2A–H2B dimers. Indeed, when titrating salt (NaCl) into the nucleosome solution, we observed a sigmoidal decay in the FRET intensity. To quantify nucleosome stability, we used the concentration of salt at the transition midpoint of the titration curve, i.e. the *C*_1/2_ value at which the FRET intensity decreased by 50%. Compared with the nucleosomes reconstituted from unmodified histone H4 (*C*_1/2_ = 0.621 M), the nucleosomes containing the H4K77succ showed a 14.8% lower C_1/2_ value (0.529 M), indicating the succinylation facilitated disassembly of the nucleosomes. This conclusion was also supported by the FRET experiment using native MMTV-B DNA, in which the MMTV-B nucleosome (*C*_1/2_ = 0.475 M) with relatively weak DNA-histone association was less stable than the Widom 601 nucleosome (*C*_1/2_ = 0.621 M). Importantly, as observed in the Widom 601 nucleosome, the succinylation of H4K77 resulted in a decreased C_1/2_ of ∼9% in the MMTV-B nucleosome (Supplementary Figure S9), indicating the destabilization effect caused by H4K77succ was not specific to the Widom 601-containing nucleosomes, but likely to be a widely existed phenomenon on other native nucleosomal DNA sequences.

To further examine the step-wise nucleosome disassembly process, we performed another two sets of FRET experiments to monitor the release of H2A–H2B dimers and (H3–H4)_2_ tetramer, respectively. The dissociation of H2A–H2B dimers from the nucleosome was monitored by the donor and acceptor fluorophores in H2B and the middle of DNA (H2B–DNA^−15^), respectively. As shown in Figure 2B, with increasing salt concentration, the H4K77succ nucleosome showed a 15.7% lower C_1/2_ value (0.562 M) compared with that of the unmodified nucleosome (0.667 M). Whereas, no difference in *C*_1/2_ value was observed between the unmodified and H4K77succ nucleosomes with the pair of FRET dyes placed at the DNA two internal positions (DNA^+42^–DNA^−52^) to monitor the release of (H3–H4)_2_ from DNA (Supplementary Figure S8) (26,27). Taken together, these two sets of FRET experiments suggest that the succinylation facilitates the dissociation of the H2A–H2B dimers from the nucleosome, but does not destabilize the (H3–H4)_2_ tetramer–DNA complex. It would therefore be interesting to ask how such a modification at the H4-DNA interface could facilitate dissociation of the H2A–H2B dimers, but not the (H3–H4)_2_ tetramer.

### H4K77succ increases nucleosomal DNA accessibility

**Figure 3.**
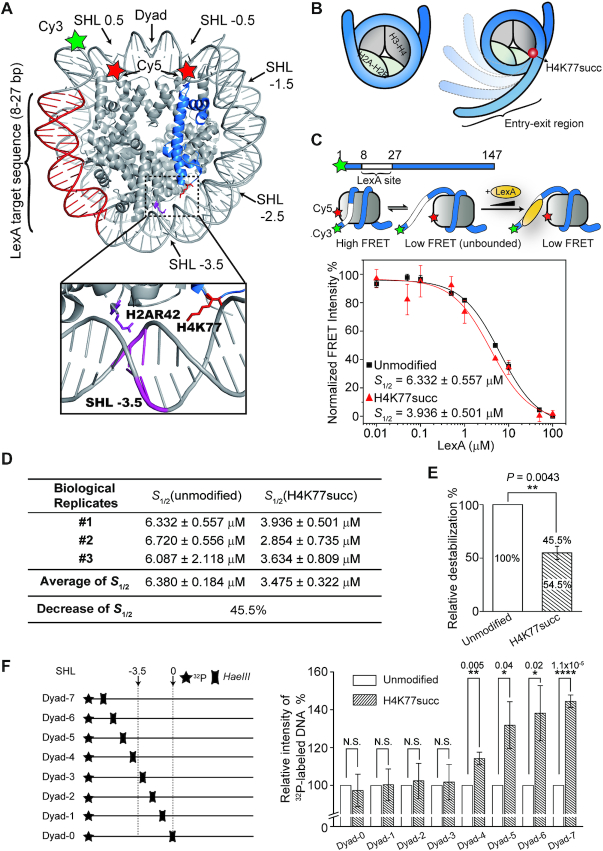
H4K77succ increased nucleosomal DNA accessibility. (**A**) Crystal structure of nucleosome (PDB: 3LZ1). The locations of Cy3/Cy5 FRET pair and LexA binding sites. The zoomed box shows the relative locations of H4K77 site and DNA SHL −3.5, which have strong ionic interaction with H2AR42 site. (**B**) Cartoons show succinylated H4K77 enhances the accessibility to nucleosomal DNA at the entry-exit region. (**C**) Upper: Diagram of DNA used to reconstitute the nucleosomes. The DNA is a 147 bp 601 nucleosome positioning sequence with a LexA binding site from 8–27 bp and labeled with Cy3 at the 5′ end; Middle: A three-state model for LexA binding to its target site within a nucleosome. Lower: Plot shows the normalized FRET intensity as a function of LexA concentration for unmodified H4 (black) and H4K77succ (red) nucleosomes. The LexA concentration at which the FRET decreases by 50% is denoted as *S*_1/2_. For visualization, all curves were normalized between 100% at the maximum FRET value and 0 at highest LexA concentration. Titration was repeated three times for each type of nucleosome (*n* = 3, *S*_1/2_ = mean ± s.e.m.). (**D**) The *S*_1/2_ values of independent experiments on each type of nucleosome are shown. The average of *S*_1/2_ (mean ± s.e.m.) was calculated from three biological replicates (independent nucleosome preparations). The decrease in *S*_1/2_ value was calculated as $( {{S_{1/2}}({\rm{unmodified}}) - {S_{1/2}}({\rm{H}}4{\rm{K}}77{\rm{succ}})} )/{S_{1/2}}({\rm{unmodified}}) \times {\rm{\ }}100{\rm{\% }}$. (**E**) The histogram shows the change in nucleosomal DNA accessibility associated with succinylation modification. The mean of the *S*_1/2_ values of unmodified nucleosomes was set to 100%. The *P* values were based on a two-tailed Student's t test. **P < 0.01. (**F**) A ‘one-pot’ assay to examine the accessibility of nucleosomal DNA. The nucleosomes were assembled with DNA containing *HaeIII* at the indicated positions. The amount of *HaeIII*-digested DNA was detected and quantified by phosphorimaging. The histogram shows the change of intensity of ^32^P-labeled DNA fragments associated with succinylation. Data are represented as mean ± s.e.m. from four independent experiments (*n* = 4). The mean of the intensities of unmodified nucleosome samples was set to 100%. The *P* values were based on a two-tailed Student's *t* test. *P < 0.05, **P < 0.01, ****P < 0.0001. N.S., no significance.

A careful structural analysis of the nucleosome revealed the sidechain of H4K77 residue is in close proximity to the superhelical location (SHL) ±3.5 of the nucleosome, which is one of known critical points of contacts between the histone octamer and nucleosomal DNA (Figure 3A). Therefore, we reasoned that H4K77succ likely impairs this key contact. As a result, the DNA at the entry/exit regions can more readily and partially unwrap away from the histone octamer (Figure 3B), which also accounts for the reduced stability in H4K77succ nucleosomes. To assess the impact of H4K77succ on DNA unwrapping, we used a previously reported FRET-based assay. This assay examines the nucleosome site accessibility by detecting the binding of a DNA-binding protein, LexA, to its target sequence buried within the nucleosome entry/exit regions (29,43).

Specifically, a LexA target sequence was inserted between 8 and 27 bp of the 147-bp 601 nucleosomal DNA. Briefly, the DNA was labeled with a Cy3 fluorophore as the FRET donor at the 5′ end near the LexA binding site, whereas the histone H2A at K119C residue was labeled with a Cy5 fluorophore as the FRET acceptor (Figure 3C, upper). Next, we titrated LexA into the FRET pair-labeled unmodified and H4K77succ nucleosomes, respectively. In the fully wrapped nucleosome, the distance between the two fluorophores was shorter than their Förster radius, which results in high FRET efficiency. With a partially unwrapped nucleosome, LexA was able to access and bind to its target site and prevent rewrapping of the nucleosome, leading to reduced FRET efficiency (Figure 3C, middle). We measured FRET efficiency in the unmodified and H4K77succ nucleosomes at various concentrations of LexA. The titration curve was then fitted to a non-cooperative binding isotherm to determine *S*_1/2_, which is concentration of LexA at which half of the nucleosomes are bound by LexA. When compared with the unmodified nucleosomes, the *S*_1/2_ value of the H4K77succ nucleosomes decreased 45.5% (Figure 3D and E). This result suggests that H4K77succ shifted the DNA wrapping-unwrapping equilibrium toward the unwrapped state, thereby increasing the accessibility of the DNA entry/exit region and exposing the histone H2A-H2B dimers. This finding was further confirmed by the ‘one-pot’ assay (31,32), in which the HaeIII restriction enzyme showed a higher digestion efficiency at dyad-4, dyad-5, dyad-6 and dyad-7 positions in H4K77succ nucleosome compared with the unmodified nucleosome (Figure 3F and Supplementary Figure S10). As dyad-4 to dyad-7 are located at the DNA terminus from SHL ±3.5, the increasement of the *Hae*III digestion efficiency on H4K77succ nucleosome revealed that the accessibility of DNA terminus was increased by H4K77succ.

### H4K77succ accelerates the nucleosome unwrapping rate

**Figure 4.**
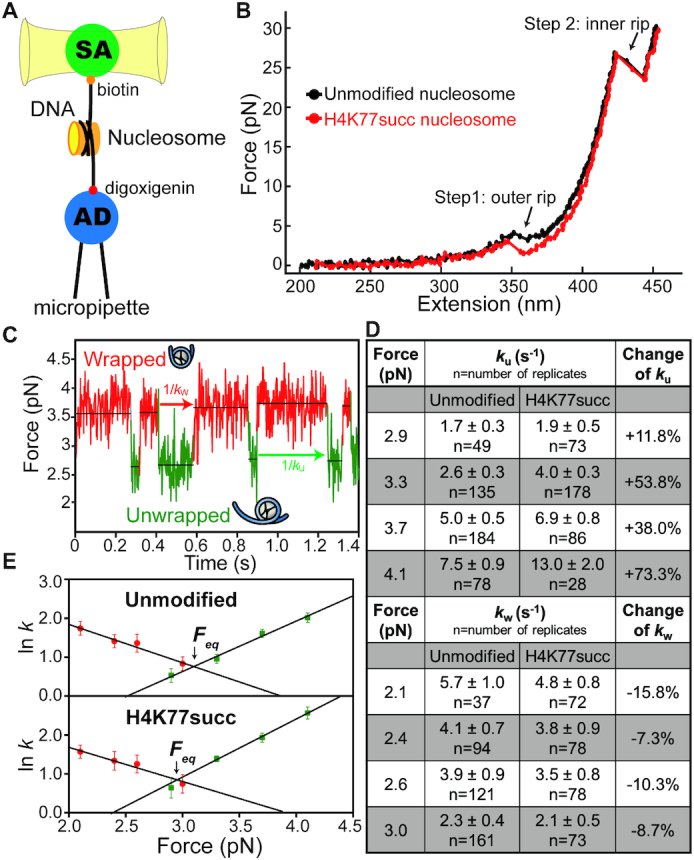
Study of single-nucleosome dynamics. (**A**) Schematic setup of the optical tweezers for studying single-nucleosome dynamics. The streptavidin-coated bead (SA) was held in the optical trap, whereas the antidigoxigenin-coated bead (AD) was held on the micropipette via suction. A single nucleosome was tethered between the beads. (**B**) Comparison of the force–extension curves of the unmodified (black) and H4K77succ (red) nucleosomes. (**C**) An example of nucleosome transition events between the wrapped and unwrapped state. (**D**) Summary of the nucleosome wrapping (*k*_w_) and unwrapping (*k*_u_) rates under the indicated external force. Mean ± s.e.m., *n* = number of replicates. (**E**) Linear fits of the natural logarithm of *k*_w_ (red dots) and *k*_u_ (green dots) versus force for unmodified and H4K77succ nucleosomes.

To further characterize the effects of H4K77succ on the DNA unwrapping rate, we used optical tweezers to monitor the real-time dynamics of individual nucleosomes under an external force (44). As shown in the experimental setup, the DNA ends of a mononucleosome were tethered on two beads, with one held in the optical trap and the other fixed on a micropipette (Figure 4A). In the experiment, the bead in the optical trap was subject to a constant pulling rate of 200 nm/s, while the force and distance between the two beads were recorded to generate a force-extension curve (Figure 4B). Consistent with previous studies, as the force increased, the DNA unwrapped from the nucleosome in two steps (44,45). In the first step occurring at a low force, the entry-exit region of DNA unwrapped and dissociated from the H2A-H2B dimers (the outer rip in Figure 4B). In the second step at a higher force, the interaction between the central region of DNA and histone (H3−H4)_2_ tetramer was disrupted (the inner rip in Figure 4B). Compared to the unmodified nucleosomes, H4K77succ nucleosomes exhibited a lower outer-rip force, indicating the Ksucc facilitated nucleosome unwrapping.

When we performed the nucleosome hopping experiment with a low force between 2 and 5 pN, the DNA outer region underwent unwrapping and rewrapping reversibly (Figure 4C), which we can use to calculate the nucleosome wrapping (*k*_w_) and unwrapping (*k*_u_) rates (Figure 4D and Supplementary Table S1). As expected, H4K77succ nucleosomes exhibited higher unwrapping rates than the unmodified nucleosomes at all applied forces. Remarkably, the H4K77succ induced greater changes in *k*_u_ at relatively higher forces (e.g. 4.1 pN) than at low forces (e.g. 2.9 pN) (Figure 4D), suggesting the unwrapping of the H4K77succ nucleosomes was more sensitive to external force changes. The natural logarithms of *k*_w_ (red dots) and *k*_u_ (green dots) were plotted against the external forces (Figure 4E). A linear fit of the plots was made to determine the equilibrium forces (*F*_eq_) at which a nucleosome had equal wrapping and unwrapping rates. The *F*_eq_ of the H4K77succ nucleosomes (2.925 pN) was lower than that of the unmodified nucleosomes (3.098 pN). The free energy cost of the H4K77succ nucleosomes outer region unwrapping at zero tension (Δ*G°*) was 16.99 kJ/mol, which was 18% lower than that of the unmodified nucleosomes (20.80 kJ/mol) (Supplementary Table S1). These results indicate H4K77succ increased the nucleosome unwrapping rate by diminishing the interactions between the nucleosomal outer region DNA and the histone octamer.

### A Ksucc mimic, H4K77E, causes defects in the chromatin structure of budding yeast

**Figure 5.**
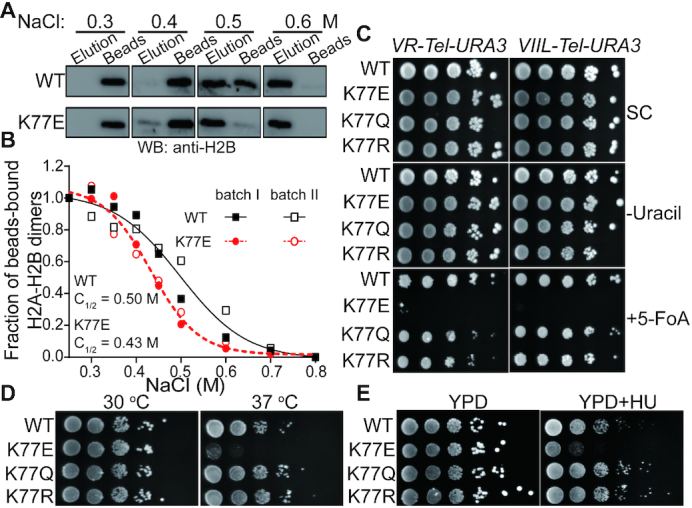
Histone H4K77E mutation reduced nucleosome stability and led to defects in chromatin compaction. In (**A**) and (**B**), the elution of histone H2A-H2B dimers from chromatin were extracted from WT and H4K77E cells. The chromatin was bound to hydroxyapatite beads and washed with the buffers containing NaCl at the indicated concentrations. The H2A-H2B dimers in the elutant and bead fractions were analyzed by Western blotting using an anti-H2B antibody. The relative amounts of the beads-bound H2A-H2B dimers (see Supplementary Figure S12) were plotted against the salt concentration in (B). Data for each batch was indicated. (**C**) *URA3* reporter gene was inserted into the indicated yeast near right arm of yeast chromosome V (*VR-Tel-URA3*) and left arm of yeast chromosome VII (*VII-Tel-URA3*). Serial dilutions of the strains bearing *URA3* were spotted on synthetic complete medium (SC), synthetic complete medium lacking uracil (–Uracil), or synthetic complete medium with 5-fluoroorotic acid (+5-FOA). The plates were incubated at 30°C for 3 days. (**D**) Serial dilutions of the indicated yeast strains were spotted on the YPD plate or YPD plus hydroxyurea (YPD+HU) plated. The plates were incubated at 30°C for 3 days. (**E**) Serial dilutions of the indicated yeast strains were spotted on the YPD plate and incubated at 30 or 37°C for 3 days.

To gain an insight into the biological functions of H4K77succ *in vivo*, we generated budding yeast strains with the histone H4K77 mutated to glutamic acid (K77E), arginine (K77R) and glutamine (K77Q), which mimic succinylated, unmodified, and acetylated lysine, respectively. We next examined changes in nucleosome stability using chromatin extracted from wild-type (WT) and H4K77E mutant yeast strains. The extracted chromatin was sheared by sonication and applied to hydroxyapatite beads, which tightly binds the chromatin through interactions between DNA and hydroxyapatite. The histone H2A–H2B dimers were eluted from the beads using buffers containing increasing salt concentrations. As shown in Figure 5A and Supplementary Figure S12, the H2A–H2B dimers from H4K77E chromatin were eluted at a lower salt concentration compared with wild-type chromatin. The fraction of beads-bound dimers was plotted against the salt concentration, and the *C*_1/2_ concentration at which half the H2A–H2B dimers were eluted was calculated by a sigmoidal fitting of the plot (Figure 5B). As expected, the *C*_1/2_ of the H4K77E chromatin (0.43 M) was significantly lower than that of the wild-type one (0.50 M), which suggested the H4K77E mutant cells have reduced nucleosome stability. It is worth mentioning that H4K77E also significantly reduced the nucleosome stability and promoted DNA unwrapping *in vitro*, which well agreed with the results for H4K77succ using the FRET and optical tweezer assays (Supplementary Figure S11).

Next, we investigated whether the impaired nucleosome stability could cause a defect in the highly compact chromatin structure of telomeres. To this end, *URA3* reporter gene was inserted near the yeast telomeres. Expression of *URA3* near telomere was assayed by comparing the viability of wild-type and H4K77 mutant cells cultured on plates containing 5-fluoro-orotic acid (5-FOA), which can be converted to cytotoxic 5-fluorouracil by *URA3* product. Figure 5C clearly shows the H4K77E mutant completely disrupted silent chromatin structure formation at the telomeres, whereas H4K77Q and H4K77R mutants had little influence on telomere silencing. This result is consistent with previous phenotypic studies of H4 K77 mutants in budding yeast (13,46), which indicates a distinct role of Ksucc in regulating the chromatin structure compared with other types of modifications at the same site. Indeed, we also observed other unique phenotypes in the K77E, but not the K77Q or H4 K77R mutant, including sensitivity to temperature (Figure 5D) and hydroxyurea treatment (Figure 5E).

In this study, we synthesized homogenous histone H4 with site-specific succinylation stoichiometrically installed at the K77 residue. This semisynthetic H4K77succ protein allowed us to reconstitute the corresponding succinylated mononucleosomes *in vitro*. We investigated the effects of Ksucc on nucleosome dynamics using various biochemical and biophysical assays. We demonstrated that this negatively charged modification decreased nucleosome stability in response to increasing ionic strength. More importantly, H4K77succ significantly accelerated the unwrapping of the outer region of the nucleosomal DNA under physiologically relevant conditions, which exposes the DNA to chromatin-binding proteins such as transcription factors.

Accumulating evidence suggests that PTMs on the globular domain of histones can serve as direct regulators of chromatin structure and dynamics. In the entry-exit region of a nucleosome, the installation of positive charge-neutralizing acetylation at lysine residues are known to regulate chromatin dynamics by interfering with histone-DNA interactions and enhance the unwrapping of DNA (47). Furthermore, lysine acetylation mapping close to the dyad axis of the nucleosome have been reported to largely attenuate the overall DNA-histone octamer interactions and nucleosome stability (9,48). Compared with lysine acetylation, histone modifications that change the net charge of the modified lysine residues from +1 to –1, for example succinylation, hold greater potential for altering nucleosome and chromatin structure and dynamics. Indeed, a recent study showed that glutarylation, another negatively charged acylation, at histone H4K91 also destabilized nucleosome structure, leading to global transcription upregulation and defects in DNA damage repair and cell-cycle progression (49). In addition to H4K77, more than 20 Ksucc sites have been identified in human nucleosomal histones (6,49), of which nine are present in histone globular domains. Comprehensive investigations of the mechanism of histone Ksucc marks and other negatively charged acylations that regulate chromatin structure and dynamics would help elucidate the functions of these modifications in chromatin biology.

## Supplementary Material

gkaa663_Supplemental_File
