# Supplementary material for: Semisynthesis of site-specifically succinylated histone reveals that succinylation regulates nucleosome unwrapping rate and DNA accessibility
Source: Nucleic Acids Res. 2020 Aug 7;48(17):9538–49. doi: 10.1093/nar/gkaa663 (PMC7515725; doi:10.1093/nar/gkaa663)
Supplement: gkaa663_Supplemental_File [file gkaa663_supplemental_file.pdf]

Supporting Information

Semisynthesis of site-specifically succinylated histone reveals that succinylation regulates nucleosome unwrapping rate and DNA accessibility

Yihang Jing, Dongbo Ding, Gaofei Tian, Ka Chun Jonathan Kwan, Zheng Liu,* Toyotaka Ishibashi,* and Xiang David Li*

List of Contents

**Figure S1**	The position of histone H4 K77 residue on nucleosome (PDB: 3LZ1).
**Figure S2**	LC-MS analysis of H4K77succ (76-102, A76C) peptide.
**Figure S3**	LC-MS and deconvolution results for the ligated product before and after desulfurization.
**Figure S4**	LC-MS and deconvolution results of purified Alexa 488-labeled histone H2B at T112 position.
**Figure S5**	LC-MS and deconvolution results of purified Cy5-labeled histone H2A at K119 position.
**Figure S6**	The succinylation on H4K77 effects nucleosome stability.
**Figure S7**	(a) The purity of nucleosomes for FRET assays. (b) The relative locations of fluorophores on nucleosomes.
**Figure S8**	FRET-based approach to study the effect of histone H4K77succ on nucleosome stability.
**Figure S9**	FRET-based approach to study the effect of histone H4K77succ on nucleosome reconstituted from MMTV-B, native positioning sequence.
**Figure S10**	The results of ‘one-pot’ assay.
**Figure S11**	FRET-based approach to study the effect of histone H4K77E on nucleosome
**Figure S12**	Monitor the amount of H2A-H2B remains on beads by Western blot.
**Table S1**	Summary of the nucleosome outer rip hopping data.
**Table S2**	Yeast strains used in this study.

Experimental Methods

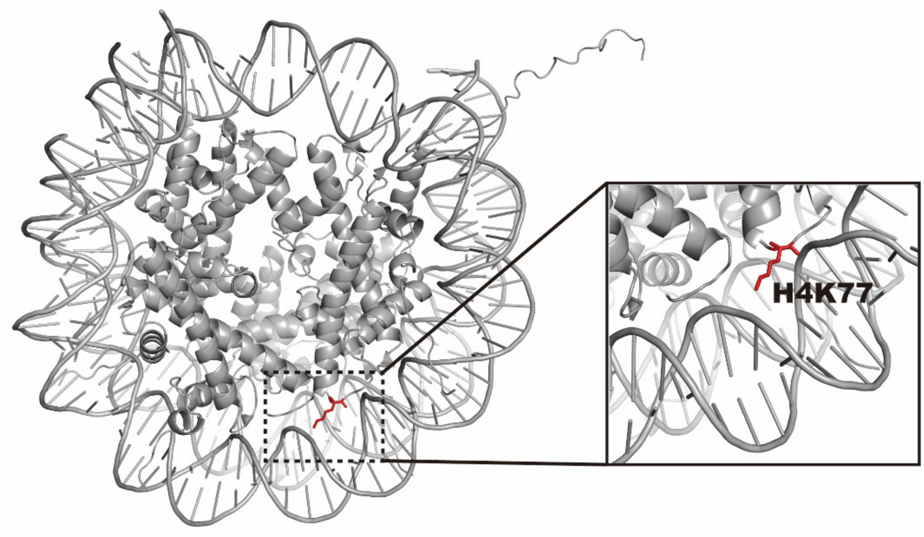

Figure S1. The position of histone H4 K77 residue on nucleosome (PDB: 3LZ1).

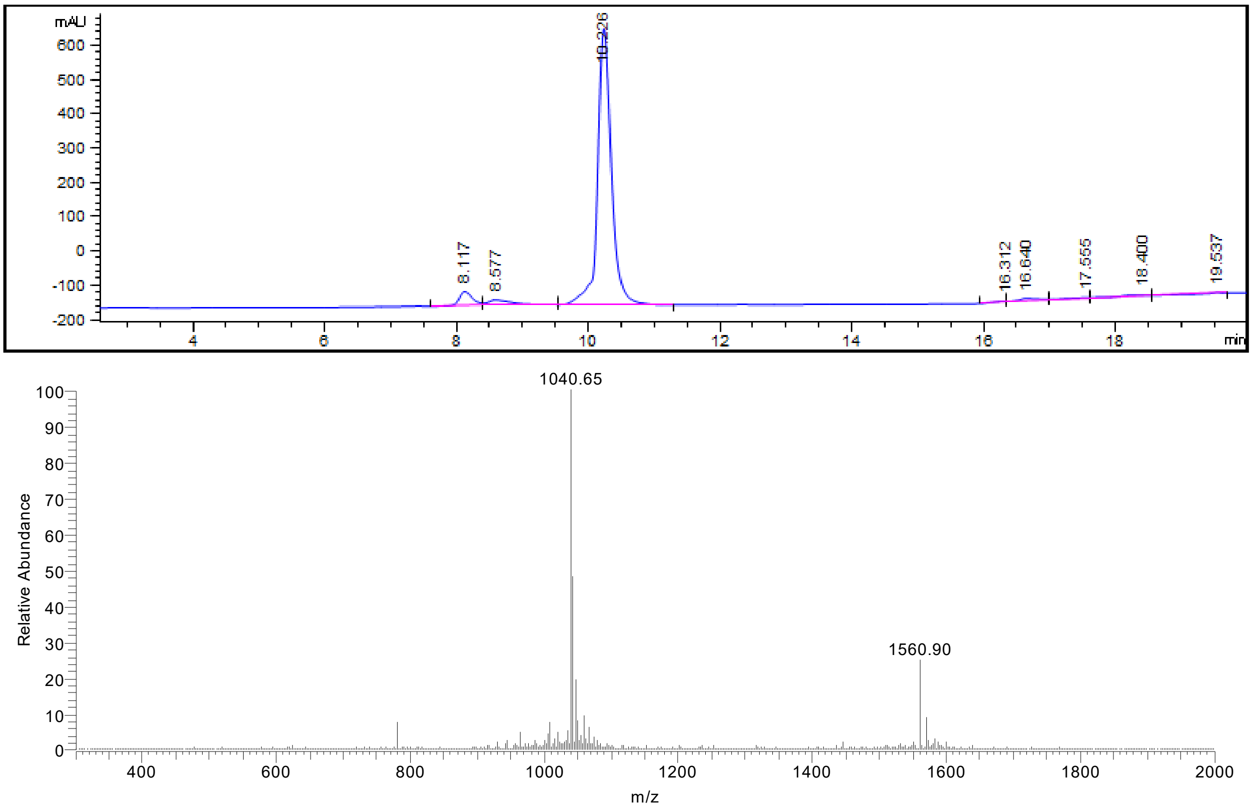

Figure S2. LC-MS analysis of H4K77succ (76-102, A76C) peptide. *m/z* 1560.90 ($M+2H^+$), calculated *m/z* 1559.81 ($M+2H^+$); *m/z* 1040.65 ($M+3H^+$), calculated *m/z* 1040.20 ($M+3H^+$). The sequence of the peptide: Cys-Lys(succ)-Arg-Lys-Thr-Val-Thr-Ala-Met-Asp-Val-Val-Tyr-Ala-Leu-Lys-Arg-Gln-Gly-Arg-Thr-Leu-Tyr-Gly-Phe-Gly-Gly.

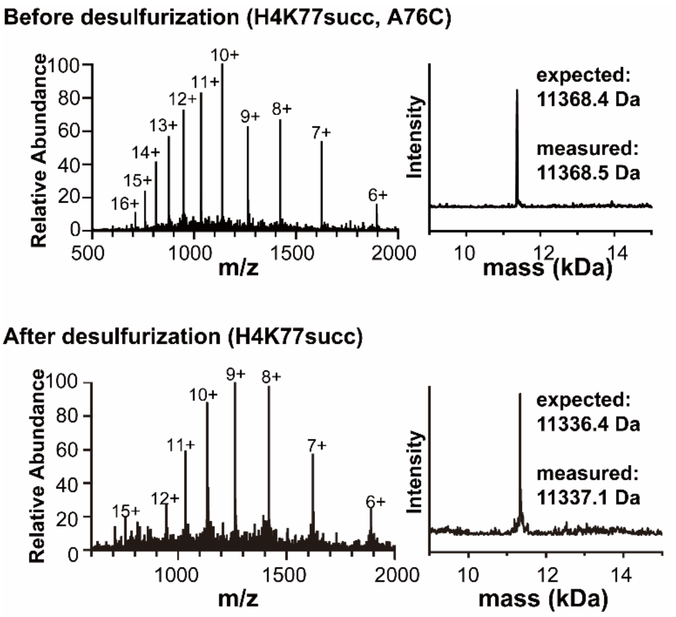

Figure S3. LC-MS and deconvolution results for the ligated product before and after desulfurization.

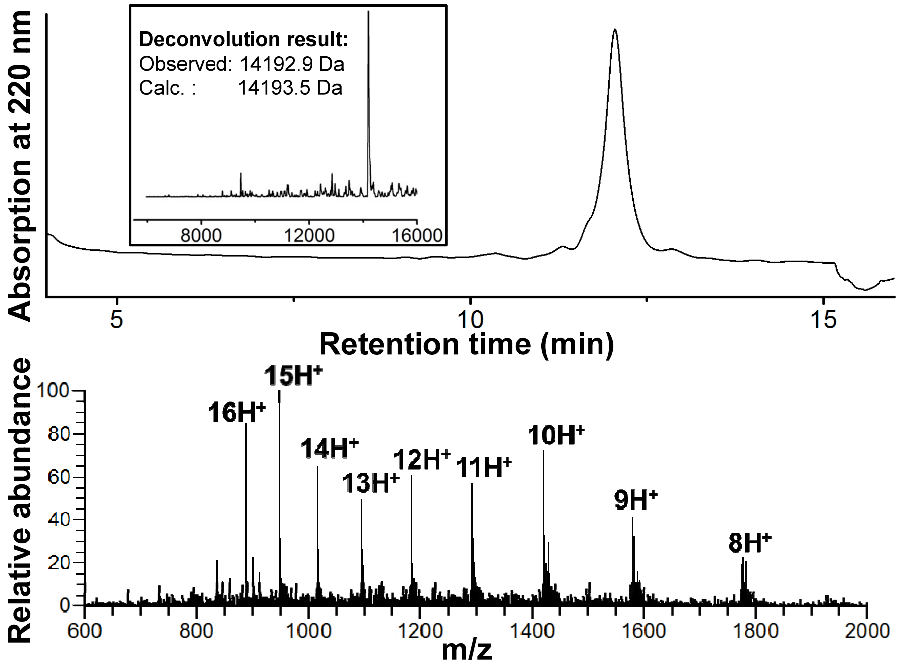

Figure S4. LC-MS and deconvolution results of purified Alexa 488-labeled histone H2B at T112 position.

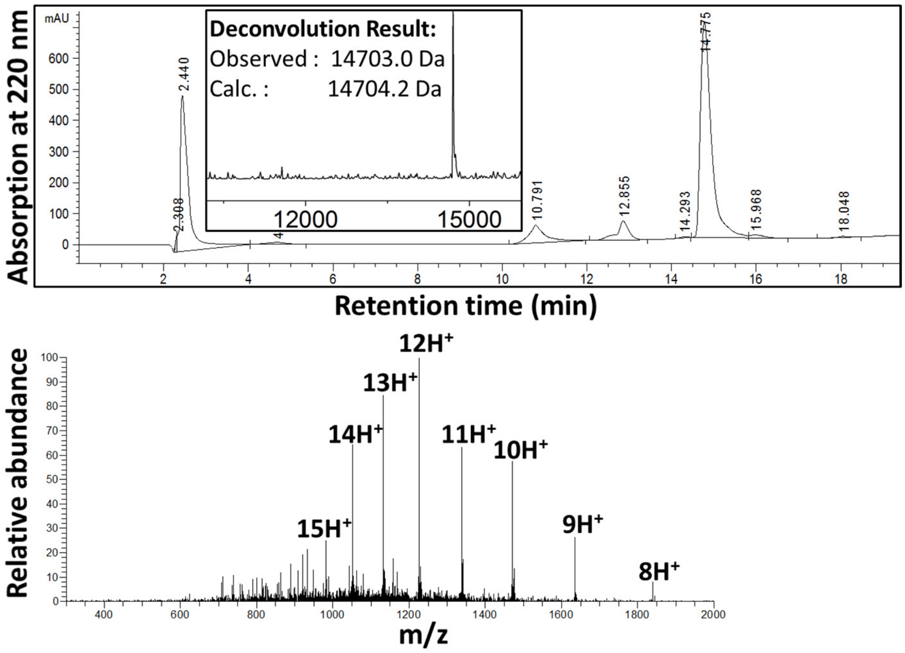

Figure S5. LC-MS and deconvolution results of purified Cy5-labeled histone H2A at K119 position.

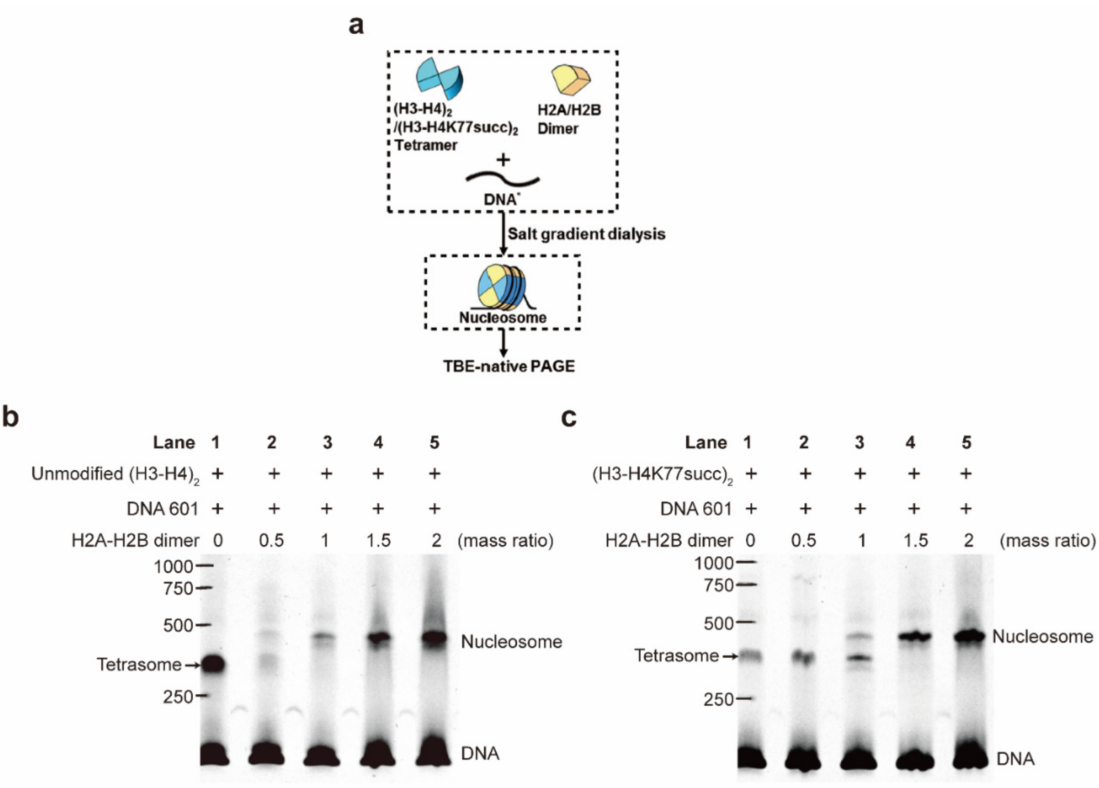

Figure S6. The succinylation on H4K77 effects nucleosome stability. (**a**) Schematic for reconstituting mononucleosomes from dimer, tetramer and DNA *in vitro*. The processed of unmodified (**b**) and H4K77succ (**c**) nucleosome assembly by adding indicated amount of H2A-H2B dimer. The nucleosome reconstituted results were analyzed by EMSA.

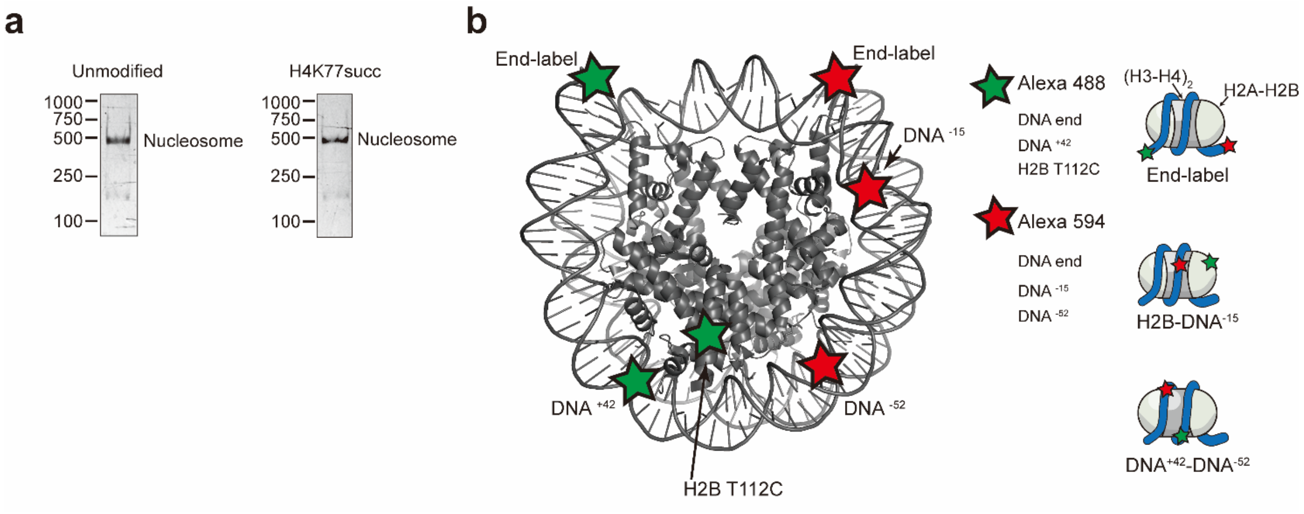

Figure S7. (**a**) A representative result of the purified unmodified and H4K77succ nucleosomes for FRET experiments. The reconstituted nucleosomes were purified by Mini Prep Cell (Model 491, Bio-rad) to remove the excess of dimer and aggregated histone-DNA complexes. The purity and quality of purified nucleosomes were checked by EMSA in 5% native polyacrylamide gel. (**b**) Crystal structure (PDB 3LZ1) and approximate dye positions for end-labeled nucleosome (End-label), internally labeled nucleosome (DNA^{+42}-DNA^{-52}) and H2B-DNA^{-15} labeled nucleosome (H2B T112-DNA^{-15}).

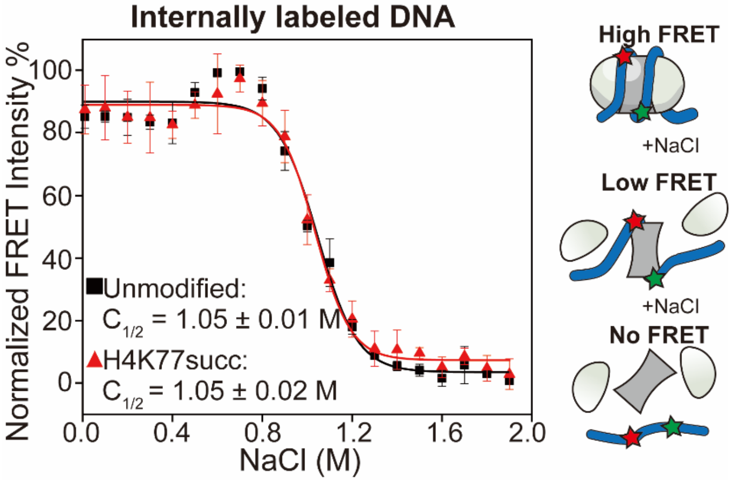

Figure S8. FRET-based approach to study the effect of histone H4K77succ on nucleosome stability. Plot showing the normalized FRET intensity versus salt concentration of unmodified (black trace) and H4K77succ (red trace) nucleosomes that labeled by FRET pair at internal DNA (DNA^{+42}-DNA^{-52}). The salt concentration at which the FRET has decreased by 50% is denoted as $C_{1/2}$. For visualization, all curves were normalized between 100% at the maximum FRET value and 0 at high salt. Salt titration

was repeated 3 times on each type of nucleosomes (n=3, $C_{1/2}$ = mean ± s.e.m).

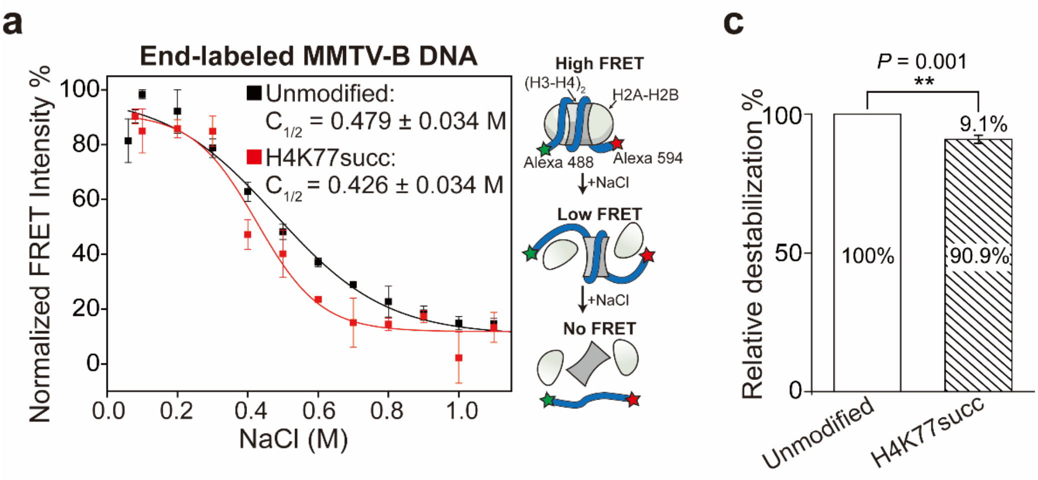

b

Biological Replicates	$C_{1/2}$(unmodified)	$C_{1/2}$(H4K77succ)
#1	0.479 ± 0.034 M	0.426 ± 0.034 M
#2	0.477 ± 0.032 M	0.430 ± 0.045 M
#3	0.469 ± 0.015 M	0.440 ± 0.039 M
Average of $C_{1/2}$	0.475 ± 0.003 M	0.432 ± 0.004 M
Decrease of $C_{1/2}$	9.1%	

Figure S9. FRET-based approach to study the effect of histone H4K77succ on nucleosome reconstituted from MMTV-B, native positioning sequence. (**a**) Plot showing the normalized FRET intensity versus salt concentration of unmodified (black trace) and H4K77succ (red trace) nucleosomes that labeled by FRET pair at DNA ends. The salt concentration at which the FRET has decreased by 50% is denoted as $C_{1/2}$. For visualization, all curves were normalized between 100% at the maximum FRET value and 0 at high salt. Salt titration was repeated 3 times on nucleosomes from one preparation (n=3, $C_{1/2}$ = mean ± s.e.m). (**b**) The $C_{1/2}$ values of independent experiments on each type of nucleosomes were showed. The average of $C_{1/2}$ (mean ± s.e.m) was calculated from 3 biological replicates (independent nucleosome preparations). (**c**) Histogram showed the change in nucleosome stability associated with succinylation modification. The mean of $C_{1/2}$ values of unmodified nucleosomes were set to 100%. The *P* values are based on the two-tailed Student's t test. **$P < 0.01$.

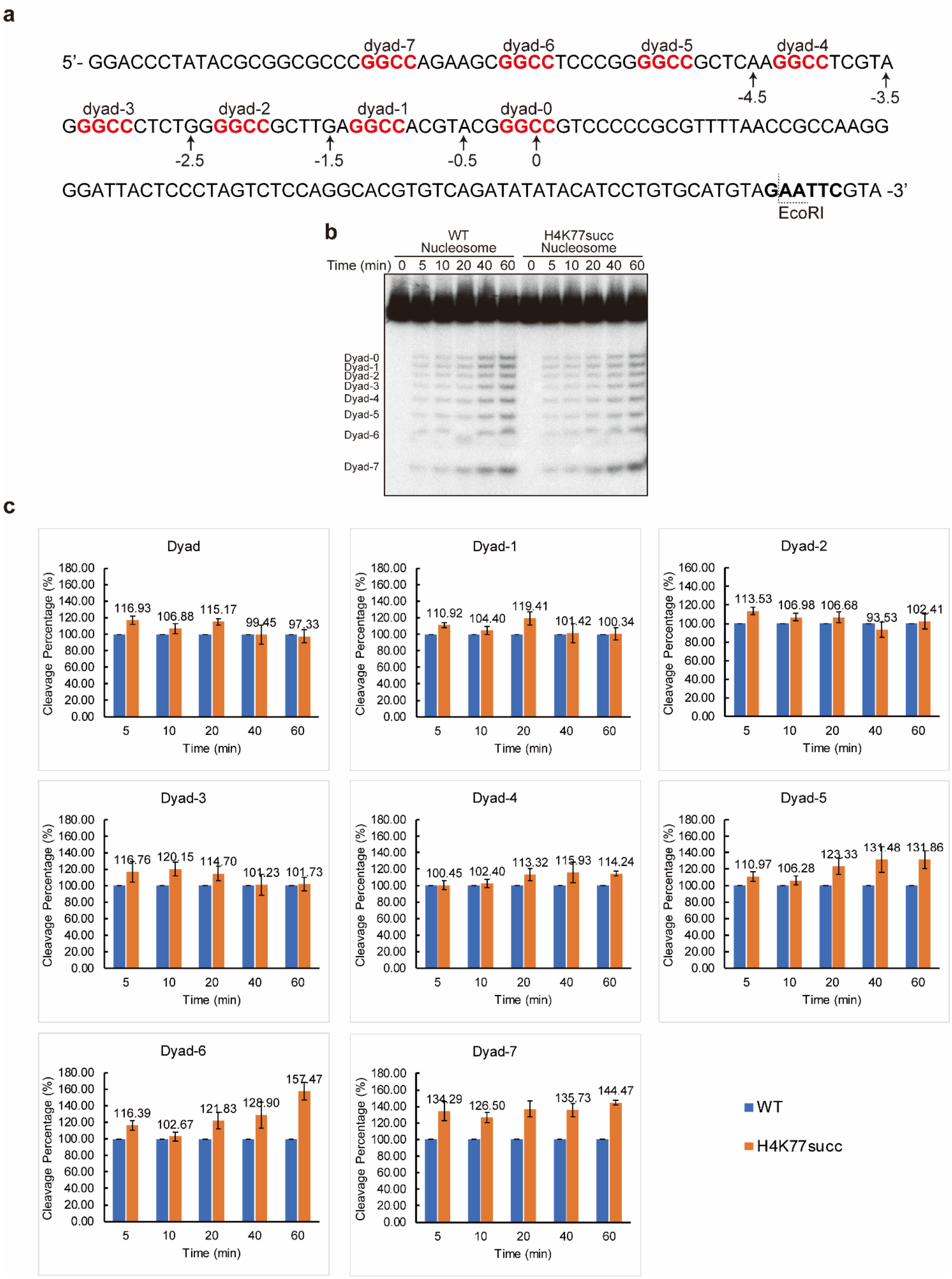

Figure S10. (a) The DNA sequence used for a ‘one-pot’ assay. (b) A representative result of HaeIII digestion. The ^{32}P-labeled DNA fragment after digestion was detected by phosphorimaging. (c) The

quantification of the HaeIII digestion on unmodified (blue) and H4K77succ (orange) nucleosome. Data was represented as mean ± s.e.m. from 4 independent experiments (n=4).

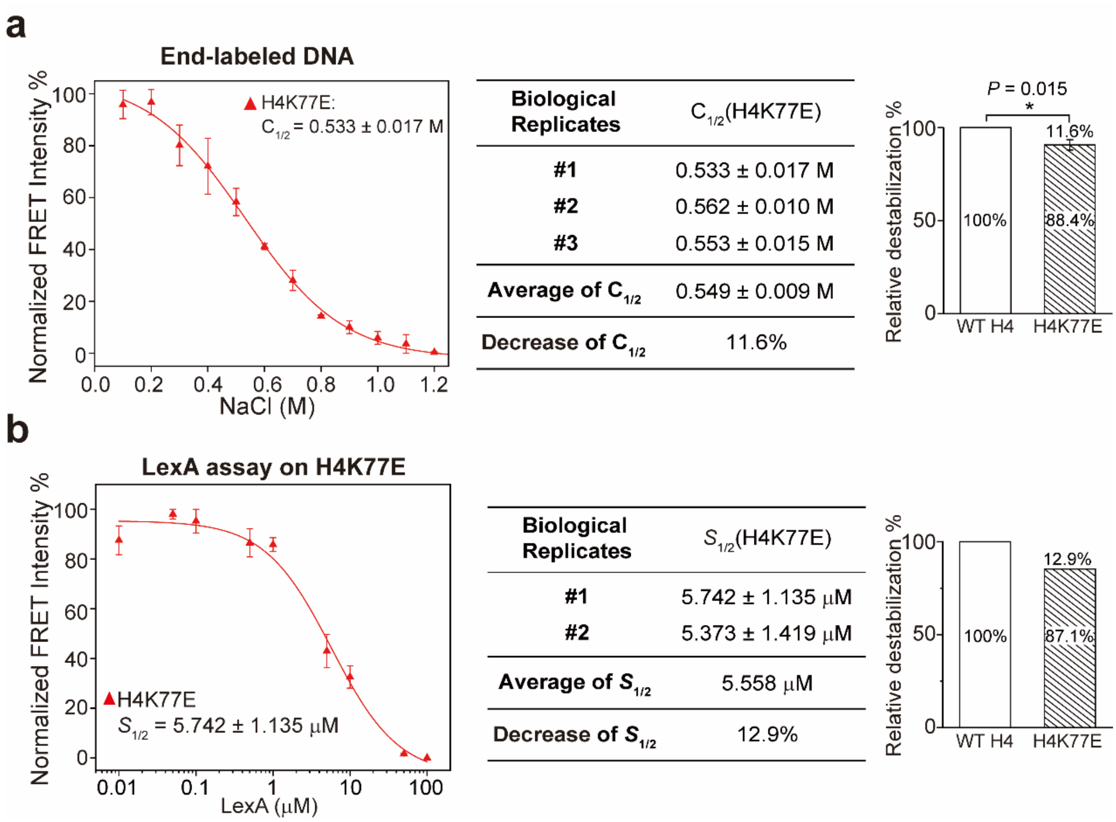

Figure S11. (**a**) Left panel, the plot showing the normalized FRET intensity versus salt concentration of H4 K77E nucleosomes that labeled by FRET pair at DNA ends. The salt concentration at which the FRET has decreased by 50% is denoted as $C_{1/2}$. For visualization, all curves were normalized between 100% at the maximum FRET value and 0 at high salt. Salt titration was repeated 3 times on nucleosomes from one preparation (n=3, $C_{1/2}$ = mean ± s.e.m). Middle panel, $C_{1/2}$ values of independent experiments on H4 K77E nucleosomes were showed. The average of $C_{1/2}$ (mean ± s.e.m) was calculated from 3 biological replicates (independent nucleosome preparations). Left panel, histogram showed the change in nucleosome stability associated with K-to-E mutation. The mean of $C_{1/2}$ values of unmodified nucleosomes were set to 100%. The *P* values are based on the two-tailed Student's t test. *P < 0.05. (**b**) Left panel, plot showing the normalized FRET intensity as a function of LexA concentration for the nucleosomes containing H4 K77E mutation. The LexA concentration at which the FRET has decreased by 50% is denoted as $S_{1/2}$. For visualization, all curves were normalized

between 100% at the maximum FRET value and 0 at high concentration of LexA. Titration was repeated 3 times on nucleosomes from one preparation (n=3, $S_{1/2}$ = mean ± s.e.m). Middle panel, the $S_{1/2}$ values of independent experiments on H4 K77E nucleosomes were showed. The average of $S_{1/2}$ was calculated from 2 biological replicates (independent nucleosome preparations). Right panel, histogram showed the change in nucleosomal DNA accessibility associated with K-to-E mutation. The mean of $S_{1/2}$ values of unmodified nucleosomes were set to 100%.

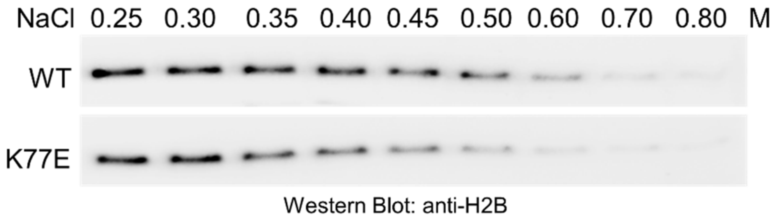

Figure S12. Monitor the amount of H2A-H2B remains on beads by Western blot. An equal amount of chromatin from wild-type and H4 K77E cells was applied to hydroxyapatite beads, respectively. Then, the beads were treated with buffers containing different concentrations of NaCl to elute the dimers. The beads were then heated in SDS loading buffer for SDS-PAGE and Western blot analysis. The histone H2B was detected by Western blot using anti-H2B. The representative result from two repeats.

Table S1. Summary of the nucleosome outer rip hopping data.

	N (Unwrap/Rewrap)	Mean hopping size (nm)	F_{eq} (pN)	Energy barrier (kJ/mol)
**Unmodified**	446/413	20.93±0.70	3.098	20.80
**H4K77succ**	365/301	20.96±0.68	2.925	16.99

Table S2. Yeast strains used in this study.

Name	Genotype	Source
YPH499	*MAT a met15-Δ0 ura3-52 lys2-801 ade2-101 his3-Δ200 trp1-Δ63 leu2-Δ1*	Dr. Yuen, Karen W.Y.
XDL16	*MAT a met15-Δ0 ura3-52 lys2-801 ade2-101 his3-Δ200 trp1-Δ63 leu2-Δ1, VIIL::URA3-TEL*	This paper
XDL18	*MAT a met15-Δ0 ura3-52 lys2-801 ade2-101 his3-Δ200 trp1-Δ63 leu2-Δ1, VR::URA3-TEL*	Dr. Xiang David Li
XDL19	*MAT a met15-Δ0 ura3-52 lys2-801 ade2-101 his3-Δ200 trp1-Δ63 leu2-Δ1, hhf1-K77E::KanMX6*	This paper
XDL20	*MAT a met15-Δ0 ura3-52 lys2-801 ade2-101 his3-Δ200 trp1-Δ63 leu2-Δ1, hhf1-K77Q::KanMX6*	This paper
XDL21	*MAT a met15-Δ0 ura3-52 lys2-801 ade2-101 his3-Δ200 trp1-Δ63 leu2-Δ1, hhf1-K77R::KanMX6*	This paper
XDL23	*MAT a met15-Δ0 ura3-52 lys2-801 ade2-101 his3-Δ200 trp1-Δ63 leu2-Δ1, hhf2Δ::TRP1 hhf1-K77E::KanMX6*	This paper
XDL24	*MAT a met15-Δ0 ura3-52 lys2-801 ade2-101 his3-Δ200 trp1-Δ63 leu2-Δ1, hhf2Δ::TRP1 hhf1-K77Q::KanMX6*	This paper
XDL25	*MAT a met15-Δ0 ura3-52 lys2-801 ade2-101 his3-Δ200 trp1-Δ63 leu2-Δ1, hhf2Δ::TRP1 hhf1-K77R::KanMX6*	This paper

XDL27	*MAT a met15-Δ0 ura3-52 lys2-801 ade2-101 his3-Δ200 trp1-Δ63 leu2-Δ1, hhf2Δ::TRP1 hhf1-K77E::KanMX6, VR::URA3-TEL*	This paper
XDL28	*MAT a met15-Δ0 ura3-52 lys2-801 ade2-101 his3-Δ200 trp1-Δ63 leu2-Δ1, hhf2Δ::TRP1 hhf1-K77Q::KanMX6, VR::URA3-TEL*	This paper
XDL29	*MAT a met15-Δ0 ura3-52 lys2-801 ade2-101 his3-Δ200 trp1-Δ63 leu2-Δ1, hhf2Δ::TRP1 hhf1-K77R::KanMX6, VR::URA3-TEL*	This paper
XDL31	*MAT a met15-Δ0 ura3-52 lys2-801 ade2-101 his3-Δ200 trp1-Δ63 leu2-Δ1, hhf2Δ::TRP1 hhf1-K77E::KanMX6, VIIL::URA3-TEL*	This paper
XDL32	*MAT a met15-Δ0 ura3-52 lys2-801 ade2-101 his3-Δ200 trp1-Δ63 leu2-Δ1, hhf2Δ::TRP1 hhf1-K77Q::KanMX6, VIIL::URA3-TEL*	This paper
XDL33	*MAT a met15-Δ0 ura3-52 lys2-801 ade2-101 his3-Δ200 trp1-Δ63 leu2-Δ1, hhf2Δ::TRP1 hhf1-K77R::KanMX6, VIIL::URA3-TEL*	This paper

Experimental Methods

Site-directed mutagenesis on histone H2A and H2B.

The cysteine mutation on histone H2B threonine 112 (H2B T112C) was introduced by site-directed mutagenesis using the following primers:

Forward, 5'-CGCCGTGTCCGAGGGCTGCAAGGCTGTCACCAAG-3';

Reverse, 5'-CTTGGTGACAGCCTTGCAGCCCTCGGACACGGCG-3';

The cysteine mutation on histone H2A lysine 119 (H2A K119C) was introduced by site-directed mutagenesis using the following primers:

Forward, 5'-CGTGCTGCTGCCCAAGTGCACCGAGAGTTCCAAGTC-3';

Reverse, 5'-GACTTGGAACTCTCGGTGCACTTGGGCAGCAGCACG-3';

All the recombinant histones (histone H1.4, H2A, H2B, H3, H4, H2BT112C, H2AK119C) were expressed in an *E. coli* strain Rosetta (DE3) and purified as previously described(1).

H4(1-75) thioester production.

Truncated histone H4 (residues 1-75) was cloned as a fusion protein with the intein and a chitin binding domain (CBD) into the pTXB1 vector (New England Biolabs). The primers were used to generate H4(1-75) fragment:

Forward 5'-GTCATATGTCTGGTCGTGGTAAAGGTGGTAAAGG-3'

Reverse 5'-CAGCTCTTCCGCAGTGTTCGGTGTAGGTAACAGCGTCAC-3'

Then the PCR product was digested with NdeI and SapI and ligated with predigested pTXB1 vector to afford plasmid of pTXB1-H4(1-75)-intein-CBD.

The protein was expressed in *Escherichia coli* Rosetta (DE3) cells and purified from inclusion bodies. The purified protein of H4(1-75)-intein-CBD was refolded by dialyzing into a high salt buffer (1 M NaCl, 25 mM HEPES, pH 7.5, 1 mM EDTA) at 4 °C. Thiolysis was initiated by adding 2-mercaptoethanesulfonate (MESNA) to a final concentration of 100 mM. Keep at 4 °C for 24 h. The cleavage yield was checked by running 15% SDS-PAGE gel. After the thiolysis, the buffer components were adjusted to ligation buffer (6 M guanidine, 25 mM HEPES, pH 7.5, 1 M NaCl, 1 mM EDTA, 50 mM MESNA) and the proteins were concentrated to >1 mg/ml and stored at -80 °C.

Expressed protein ligation to prepare modified histone H4K77succ with A76C mutation.

10 molar equivalents of H4K77succ (76-102, A76C) peptide was added to the H4 (1-75) thioester solution in ligation buffer. Tris(2-carboxyethyl)phosphine (TCEP) was added to a final concentration of 20 mM, and the pH of the ligation mixture was adjusted to 7.5 by using 1 M NaOH. The ligation reaction was allowed to proceed

for 24-48 h at 4 °C. LC-MS was applied to monitor the ligation reaction.

Desulfurization of Cys to native Ala residue.

TECP (1.0 M stock in H_2O) was added into the crude ligation mixture to a final concentration of 300 mM. MESNA was added to a final concentration of 50 mM. The reaction solution was degased for 30 min. Add 2,2'-azobis[2-(2-imidazolin-2-yl) propane] dihydrochloride (VA-044) to a final concentration of 10 mM and incubate at 42 °C until the reaction is completed as monitored by LC-MS. Then the histone H4K77succ product was purified by HPLC using preparative C4 column (22mm X 250 mm, Grace) in a gradient of 30-60% buffer B in 40 min. The purified product was confirmed by LC-MS. Deconvolution result was obtained by UniDec software(2).

Western blotting

The samples were separated by 15% SDS-PAGE and then were transferred onto a polyvinylidene fluoride (PVDF) membrane followed by blocking in TBST buffer (0.1% Tween-20 in TBS buffer) containing 5% nonfat-dried milk for 1 h at room temperature. The membrane was then incubated with primary antibody diluted in TBST buffer with 5% nonfat-dried milk for overnight at 4 °C, followed by washing with TBST buffer for twice, each time 15 min. After incubating with secondary antibody diluted in TBST buffer with 5% BSA for 1 h at room temperature, the membranes were washed with TBST for 4 times, 10 min each, and visualized with SuperSignal West Pico or Dura Chemiluminescent Substrate by a MyECL Imager system (Thermo Fisher Scientific).

Reference

1. Luger, K., Rechsteiner, T.J., and Richmond, T.J. (1999) Expression and purification of recombinant histones and nucleosome reconstitution. *Methods in molecular biology*. **119**, 1-16.
2. Marty, M.T., Baldwin, A.J., Marklund, E.G., Hochberg, G.K., Benesch, J.L., and Robinson, C.V. (2015) Bayesian deconvolution of mass and ion mobility spectra: from binary interactions to polydisperse ensembles. *Analytical chemistry*. **87**, 4370-4376.
